# Fractalkine/CX3CL1 induced intercellular adhesion molecule-1-dependent tumor metastasis through the CX3CR1/PI3K/Akt/NF-κB pathway in human osteosarcoma

**DOI:** 10.18632/oncotarget.11250

**Published:** 2016-08-12

**Authors:** Ju-Fang Liu, Ya-Ting Tsao, Chun-Han Hou

**Affiliations:** ^1^ Central Laboratory, Shin-Kong Wu Ho-Su Memorial Hospital, Taipei, Taiwan; ^2^ Department of Orthopedic Surgery, National Taiwan University Hospital, Taipei, Taiwan

**Keywords:** fractalkine, osteosarcoma, metastasis, cell migration, ICAM-1

## Abstract

Osteosarcoma is the most common primary bone tumor in children and teens. The exact molecular mechanism underlying osteosarcoma progression still remains unclear. The CX3CL1/fractalkine has been implicated in various tumors but not in osteosarcoma. This study is the first to show that fractalkine promotes osteosarcoma metastasis by promoting cell migration. Fractalkine expression was higher in osteosarcoma cell lines than in normal osteoblasts. Fractalkine induced cell migration by upregulating intercellular adhesion molecule-1 (ICAM-1) expression via CX3CR1/PI3K/Akt/NF-κB pathway in human osteosarcoma cells. Knockdown of fractalkine expression markedly inhibited cell migration and lung metastasis in osteosarcoma. Finally, we showed a clinical correlation between CX3CL1 expression and ICAM-1 expression as well as tumor stage in human osteosarcoma tissues. In conclusion, our results indicate that fractalkine promotes cell migration and metastasis of osteosarcoma by upregulating ICAM-1 expression. Thus, fractalkine could serve a novel therapeutic target for preventing osteosarcoma metastasis.

## INTRODUCTION

Osteosarcoma is the most common primary bone tumor in children and adolescents. Although the cause of osteosarcoma is still unknown, radiation and Paget disease may contribute to the initiation of this tumor [1, 2]. Currently, routine treatment involves surgery and chemotherapy as adjuvant therapy for preventing metastasis. However, local recurrence, distant bone invasion, or lung metastasis contribute to mortality [3–5]. Thus, novel therapy that effectively prevents metastasis from the local site of osteosarcoma is urgently required.

Cancer metastasis is a complicated process that contributes to mortality among patients with cancer. In metastasis progression, expression of cell adhesion molecules (CAMs) that promote cell–extracellular matrix (ECM) adhesion is crucial for the efficient spread of metastatic cells [6–8]. Intracellular adhesion molecule-1 (ICAM-1, also known as CD54) is a surface glycoprotein that belongs to the immunoglobulin supergene family and participates in cell–ECM adhesion [9–11]. ICAM-1 has been proposed to promote migration of many cell types [12–14]. Upregulation of ICAM-1 has also been implicated in the metastasis of many tumors. For example, knockdown of ICAM-1 inhibits invasion of prostate cancer cells [15]. ICAM-1 expression is associated with the migration and invasion of breast, lung, gastric, oral, and bone cancer cells [12, 15–18]. Furthermore, blocking ICAM-1 mRNA by using antisense oligonucleotides abolishes lung metastasis in melanoma [19]. ICAM-1 is positively related to the progression, occurrence, and metastasis of hepatocellular carcinoma [20]. Therefore, ICAM-1 is a potential therapeutic target for bone tumors.

To date, fractalkine is the only member of the CX3C chemokine family. It is expressed in many cell types such as hematopoietic cells, endothelial cells, epithelial cells, smooth muscle cells, dendritic cells, neurons, and fibroblasts [21–26]. Fractalkine exists in two forms that have distinct cellular functions. The soluble form is a chemoattractant for cells expressing the CX3CR1 receptor. The membrane-bound form functions as an adhesion molecule that promotes adhesion and migration [27]. Recently, fractalkine/CX3CR1 expression has been associated with many cancers, such as breast, colorectal, prostate, and gastric cancers [28–31]. Nevertheless, the role of fractalkine in tumor progression is controversial in the context of the cancer type and the tumor microenvironment.

Previous reviews have showed that fractalkine promotes tumor progression. Knockdown of fractalkine reduces angiogenesis in hepatocellular carcinoma [32]. Upregulation of fractalkine in high-grade gliomas is correlated with poor prognosis [33]. The fractalkine/CX3CR1 axis has been proposed to increase the metastasis of prostate cancer and tumor growth in epithelial ovarian cancer by activating Akt signaling [30, 34]. Nonetheless, the role of fractalkine in osteosarcoma is still unknown. This study is the first to provide evidence indicating that fractalkine promotes cell migration and contributes to lung metastasis by upregulating ICAM-1 expression. In addition, fractalkine-induced ICAM-1 expression and cell migration are activated by the CX3CR1/PI3K/Akt/NF-κB pathway. We found that knockdown of fractalkine inhibited lung metastasis in osteosarcoma. Moreover, IHC results show fractalkine expression is positively correlated with ICAM-1 expression and tumor stage in osteosarcoma. In summary, our results indicate that fractalkine plays a key role in the metastasis of osteosarcoma.

## RESULTS

### High expression of fractalkine is associated with migration potential in osteosarcoma

**Figure 1:**
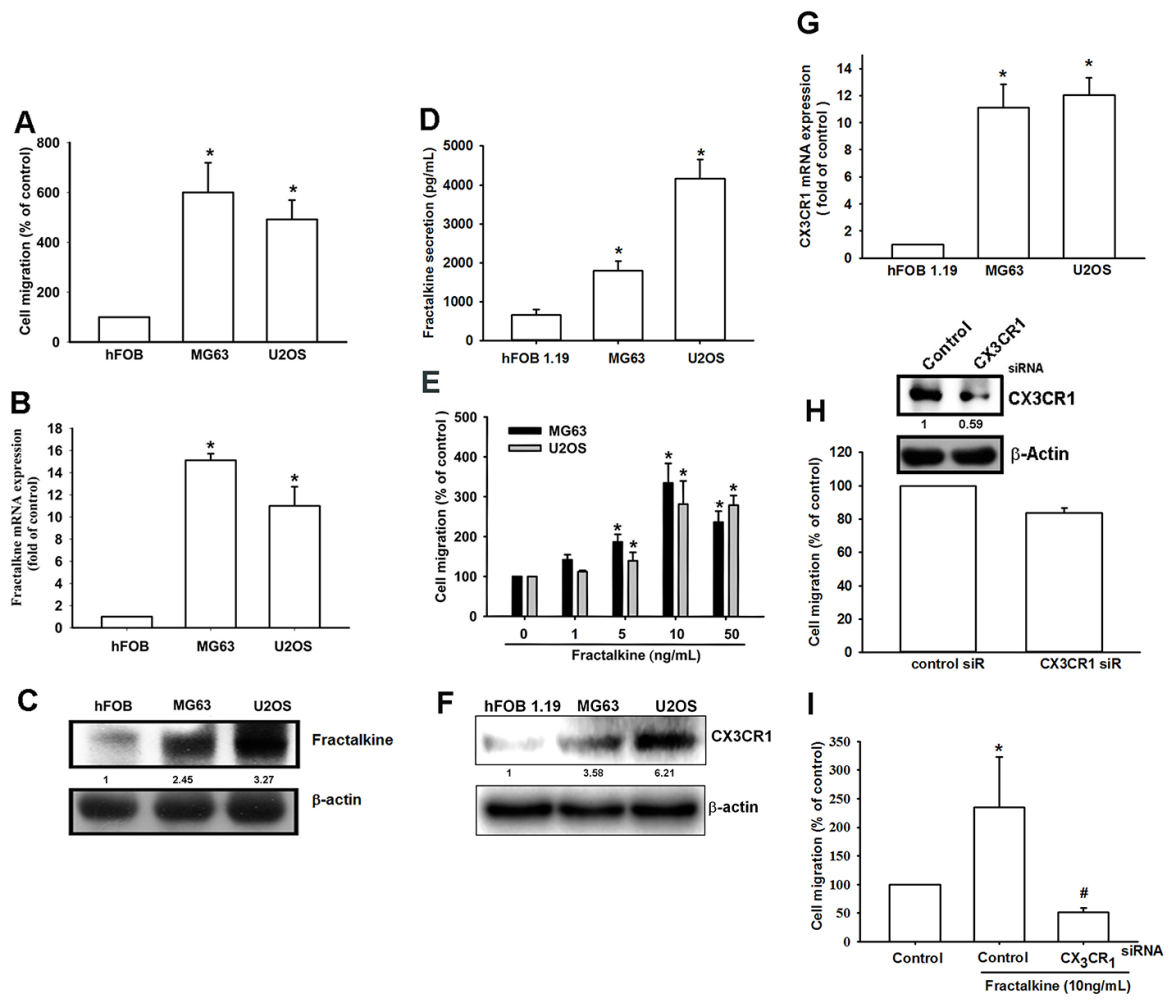
High fractalkine/CX3CL1 expression is correlated with tumor stage and cell migration ability **A**. The cell migration ability of the osteoblast cell line hFOB 1.19 and the osteosarcoma cell lines MG63 and U2OS was assessed using the Transwell assay. **B** and **D**. Total mRNA and protein were collected from the indicated cell lines, and fractalkine expression was detected using qPCR, Western blotting and ELISA assay. **E**. The osteosarcoma cell lines MG63 and U2OS were incubated with the indicated concentrations of fractalkine for 24 h, and cell migration ability was assessed using the Transwell assay. **F-G**. Total mRNA and protein were collected from the indicated cell lines, and CX3CR1 expression was detected using qPCR and Western blotting. **H-I**. MG63 cells were transfected with CX3CR1 or negative siRNA (control) for 24 h and then incubated with fractalkine (10 ng/mL) for 24 h. Cell migration ability was analyzed using the Transwell assay. Results are expressed as the mean ± SEM of triplicate samples. A-D: *P < 0.05 compared with hFOB 1.19 cells. E–I: *P < 0.05 compared with the control group and ^#^P < 0.05 compared with the fractalkine-treated group.

Previous studies have indicated that fractalkine/CX3CL1 expression is related with the progression of many cancers. However, the role of fractalkine in osteosarcoma is poorly understood. Therefore, we first assessed the correlation between fractalkine expression and the migration ability of osteosarcoma cells. The Transwell cell migration assay, qPCR, and Western blot assay indicated a positive correlation of fractalkine mRNA and protein expression with cell migration ability in the osteosarcoma cell lines (MG63 and U2OS) and normal osteoblast cell line (hFOB 1.19) (Figure 1A–1D). Cells from both osteosarcoma lines had higher fractalkine expression and cell migration ability. In addition, osteosarcoma cells treated with fractalkine had increased migration ability (Figure 1E). Next, to examine whether CX3CR1, the specific receptor of the ligand fractalkine, is involved in fractalkine-induced cell migration, comparison of CX3CR1 between hFOB1.19 and osteosarcoma is important. We examined the levels of CX3CR1 between hFOB 1.19 and osteosarcoma (MG63 and U2OS). The level of CX3CR1 was significantly elevated in MG63 and U2OS cell lines compared with hFOB 1.19 (Figure 1F-1G). To confirm this finding, MG63 cells were transfected with control and CX3CR1 small interfering RNA (siRNA) for 24 h, and the western blot analysis showed that the expression of protein levels of CX3CR1 was suppressed by transfection with CX3CR1 siRNA (Figure 1H). Transfected cells with CX3CR1 siRNA slightly reduced cell migration (Figure 1H) and significantly abolished fractalkine-induced cell migration (Figure 1I). These results indicate that the fractalkine/CX3CR1 axis plays a crucial role in the migration ability of osteosarcoma cells.

### Fractalkine-induced cell migration is mediated by ICAM-1 expression

**Figure 2:**
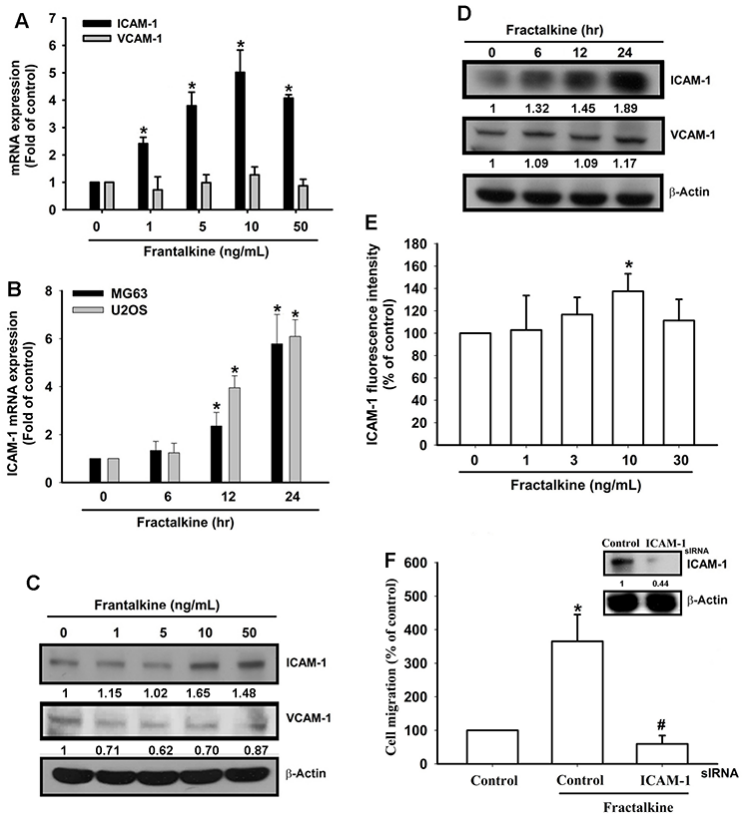
Fractalkine-induced cell migration is mediated by ICAM-1 expression in osteosarcoma **A**. MG63 cells were treated with various concentrations of fractalkine for 24 h. Total RNA was extracted, and ICAM-1 mRNA expression was measured using qPCR. **B**. The osteosarcoma cell lines MG63 and U2OS were incubated with 10 ng/mL fractalkine for the indicated times. ICAM-1 mRNA expression was measured using qPCR. **C**. MG63 cells were treated as indicated (Figure 2A), and total protein was collected. VCAM-1 and ICAM-1 expression levels were assessed using Western blotting. **D**. MG63 cells were treated as indicated (Figure 2B), and total protein was collected. The VCAM-1 and ICAM-1 expression levels were assessed using Western blotting. **E**. MG63 cells were treated with the indicated concentrations of fractalkine for 24 h. The cells were stained with the ICAM-1 antibody and FITC-conjugated secondary antibody, and ICAM-1 expression was assessed using flow cytometric analysis. **F**. MG63 cells were transfected with ICAM-1 or negative siRNA (control) for 24 h and subsequently incubated with fractalkine (10 ng/mL) for 24 h. Cell migration ability was analyzed using the Transwell assay. The upper panel shows the expression of ICAM-1 in siRNA-transfected cells. Results are expressed as the mean ± SEM of triplicate samples. *P < 0.05 compared with the control group and ^#^P < 0.05 compared with the fractalkine-treated group.

CAMs have been implicated in the spread of metastatic cells in recent decades [6–8]. However, the regulation of CAMs in human osteosarcoma cells is largely unknown. Therefore, we examined the expression of ICAM-1 and VCAM-1, the two well-established CAMs with a key role in tumor metastasis [20, 35]. ICAM-1 mRNA and protein expression increased after fractalkine treatment in a dose- and time-dependent manner (Figure 2A-2D). However, VCAM-1 expression did not change after fractalkine treatment. The flow cytometric assay showed induction of ICAM-1 in the cell membrane after fractalkine treatment (Figure 2E). To confirm the role of ICAM-1 in fractalkine-induced cell migration, MG63 cells were transfected with ICAM-1 siRNA. The transfected cells showed markedly inhibited fractalkine-induced cell migration (Figure 2F). These data clearly show that fractalkine-induced cell migration is mediated by ICAM-1 expression.

### PI3K/Akt signaling pathway is involved in fractalkine-induced ICAM-1 expression and cell migration

**Figure 3:**
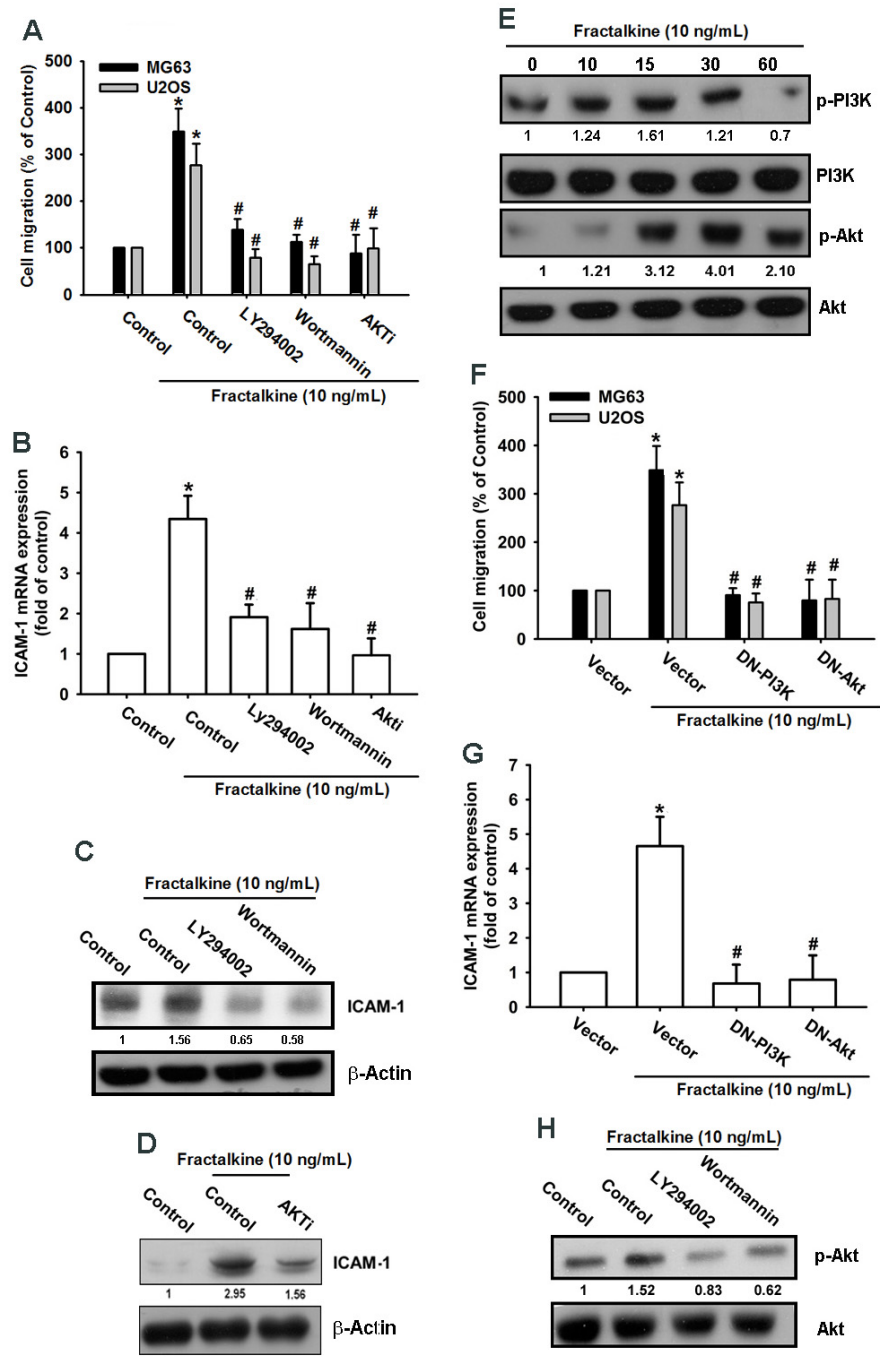
PI3K/Akt signal cascade mediates fractalkine response in human osteosarcoma **A**. The osteosarcoma cell lines MG63 and U2OS were pretreated with 0.1% DMSO as a control, LY294002 (1 μM), wortmannin (1 μM), and Akti (1 μM) for 30 min and then incubated with fractalkine for 24 h. Cell migration was examined using the Transwell assay. **B**. MG63 cells were treated as described (Figure 3A), and ICAM-1 mRNA expression was assessed using qPCR. **C** and **D**. MG63 cells were pretreated with 0.1% DMSO as a control, LY294002 (1 μM), wortmannin (1 μM), or Akti (1 μM) for 30 min and then incubated with fractalkine for 24 h. ICAM-1 protein expression was examined using Western blotting. **E**. MG63 cells were incubated with fractalkine (10 ng/mL) for the indicated time intervals. Phosphorylation of PI3K and Akt was examined using Western blotting. **F**. The osteosarcoma cell lines MG63 and U2OS were transfected with the vector control and PI3K and Akt dominant mutants (DNs) for 24 h and subsequently treated with fractalkine (10 ng/mL) for 24 h, and cell migration was analyzed using the Transwell assay. **G**. MG63 cells were treated as described (Figure 3F), and ICAM-1 mRNA expression was assessed using qPCR. **H**. MG63 cells were pretreated with 0.1% DMSO as a control, LY294002 (1 μM), and wortmannin (1 μM) for 30 min and then incubated with fractalkine for 30 min. Akt phosphorylation was examined using Western blotting. Results are expressed as the mean ± SEM of triplicate samples. *P < 0.05 compared with the control group and ^#^P < 0.05 compared with the fractalkine-treated group.

Previous studies have indicated that the fractalkine/CX3CR1 axis promotes tumor progression by activating PI3K/Akt signaling [30, 34]. In accordance with previous findings, our results indicated that pretreating cells with PI3K/Akt inhibitors (Ly294002, wortmannin, and Akti) for 30 min markedly inhibited fractalkine-induced cell migration and ICAM-1 mRNA and protein expression (Figure 3A–3D). In addition, treatment of cells with fractalkine induced phosphorylation of the PI3K p85 subunit and Akt signal proteins (Figure 3E). Transfecting osteosarcoma cells with PI3K and Akt dominant mutants (DN-PI3K and DN-Akt) also inhibited fractalkine-induced cell migration and ICAM-1 expression (Figure 3F-3G). Pretreatment with PI3K inhibitors inhibited phosphorylation of Akt, indicating that Akt is the downstream signal protein of PI3K (Figure 3H). According to these results, fractalkine induced ICAM-1 expression and cell migration through the PI3K/Akt signaling pathway in osteosarcoma.

### NF-κB signal pathway is involved in fractalkine-induced ICAM-1 expression and cell migration

**Figure 4:**
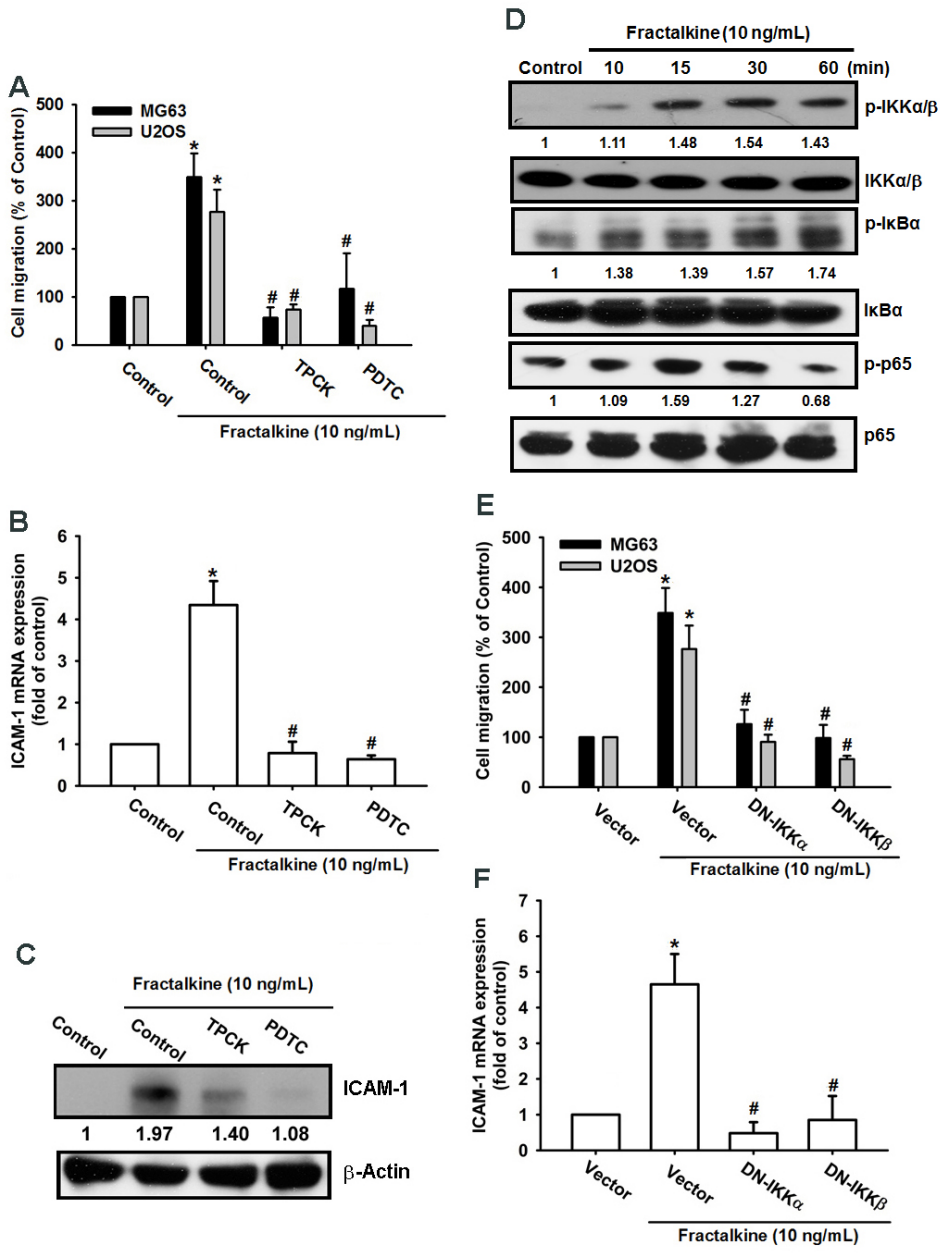
NF-κB mediates fractalkine-induced ICAM-1 expression and cell migration in human osteosarcoma **A**. The osteosarcoma cell lines MG63 and U2OS were pretreated with 0.1% DMSO as a control, PDTC (10 μM), and TPCK (1 μM) for 30 min and subsequently incubated with fractalkine for 24 h. Cell migration was examined using the Transwell assay. **B**. MG63 cells were treated as described (Figure 4A), and ICAM-1 mRNA expression was assessed using qPCR. **C**. MG63 cells were incubated with fractalkine (10 ng/mL) for the indicated time intervals. Phosphorylation of IKKα/β, IκBα, and p65 was examined using Western blotting. **D**. The osteosarcoma cell lines MG63 and U2OS were transfected with a vector control and IKKα and IKKβ dominant mutants (DNs) for 24 h and then treated with fractalkine (10 ng/mL) for 24 h. Cell migration was analyzed using the Transwell assay. **E**. MG63 cells were treated as described (Figure 4D), and ICAM-1 mRNA expression was assessed using qPCR. **F**. MG63 cells were pretreated with 0.1% DMSO as a control, PDTC (10 μM), and TPCK (1 μM) for 30 min and subsequently incubated with fractalkine for 24 h. ICAM-1 protein expression was examined using Western blotting. Results are expressed as the mean ± SEM of triplicate samples. *P < 0.05 compared with the control group and ^#^P < 0.05 compared with the fractalkine-treated group.

**Figure 5:**
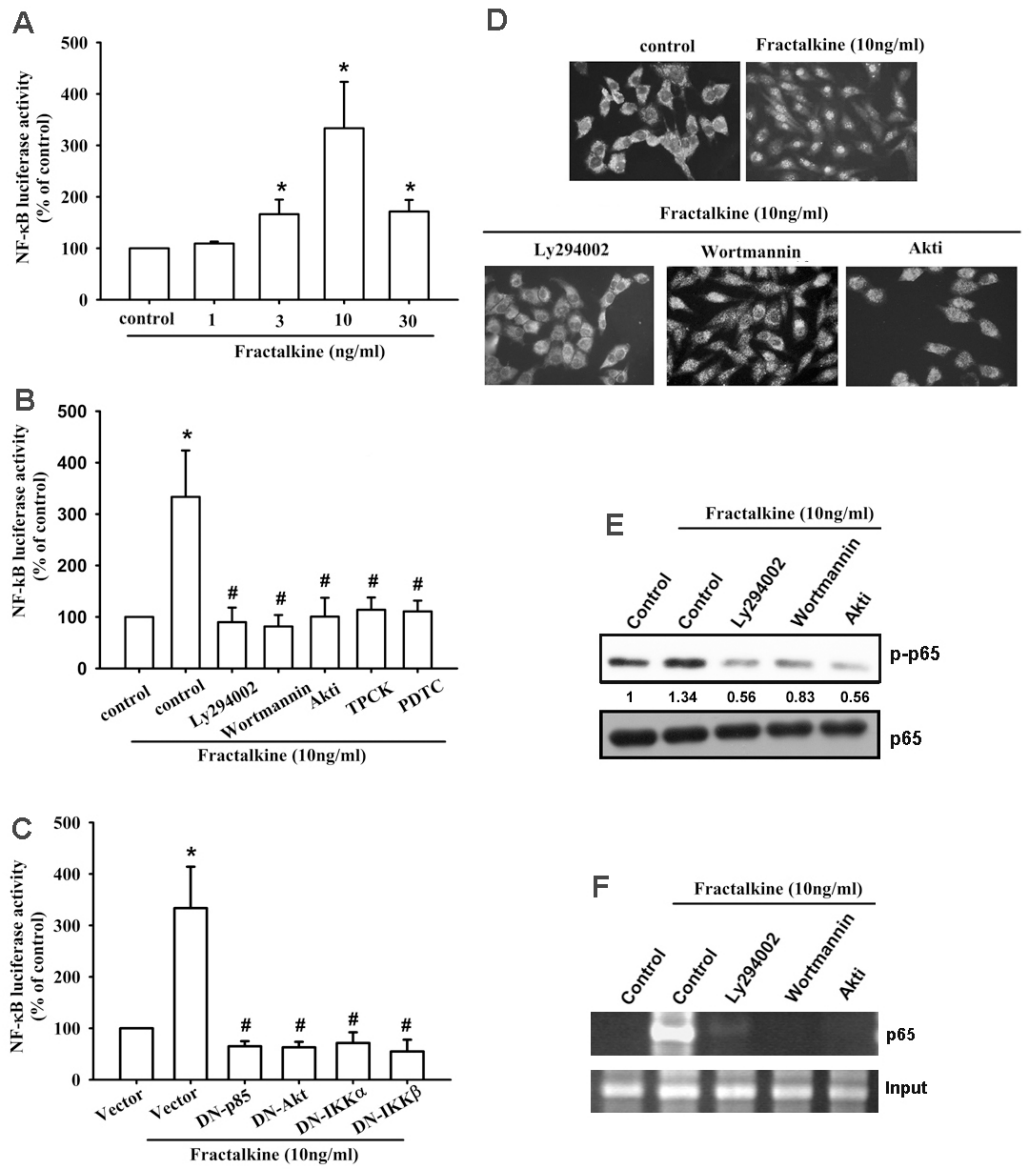
Fractalkine activates PI3K/Akt/NF-κB signal cascade in osteosarcoma **A**. MG63 cells were transfected with the NF-κB promoter reporter plasmid for 24 h and then incubated with the indicated doses of fractalkine for 24 h, and luciferase activity was measured. **B**. MG63 cells were transfected with the NF-κB promoter reporter plasmid for 24 h. Subsequently, the cells were pretreated with 0.1% DMSO as a control, LY294002 (1 μM), wortmannin (1 μM), Akti (1 μM), PDTC (10 μM), and TPCK (1 μM) for 30 min and then incubated with fractalkine (10 ng/mL) for 24 h, and luciferase activity was measured. **C**. MG63 cells were cotransfected with the NF-κB promoter reporter plasmid and p85, Akt, IKKα, and IKKβ dominant mutants for 24 h and then incubated with fractalkine (10 ng/mL) for 24 h, and luciferase activity was measured. **D**. MG63 cells were pretreated with 0.1% DMSO as a control, LY294002 (1 μM), wortmannin (1 μM), and Akti (1 μM) for 30 min and subsequently incubated with fractalkine (10 ng/mL) for 60 min. Cells were stained with the anti-p65 antibody and analyzed using fluorescence microscopy. Nuclei were counterstained with DAPI. Representative confocal microscopy images are shown. **E** and **F**. MG63 cells were treated as indicted (Figure 5D), the nuclear extracts were prepared, and phosphorylation of p65 was determined through Western blot analysis (E), or chromatin immunoprecipitation was performed with anti-p65. One percent of immunoprecipitated chromatin was assayed to verify equal loading (input) (F). Results are expressed as the mean ± SEM of triplicate samples. *P < 0.05 compared with the control group and ^#^P < 0.05 compared with the fractalkine-treated group.

A previous study indicated that NF-κB is a crucial transcription factor involved in cancer cell migration and invasion [36]. Therefore, we examined whether the NF-κB signaling pathway is involved in fractalkine-induced ICAM-1 expression and cell migration. Pretreating cells with NF-κB inhibitors (PDTC and TPCK) for 30 min markedly inhibited fractalkine-induced cell migration and ICAM-1 mRNA and protein expression (Figure 4A–4C). Moreover, treating cells with fractalkine induced phosphorylation of IKKα/β, IκB-α, and p65 signal proteins (Figure 4D). Furthermore, osteosarcoma cells transfected with IKKα and IKKβ dominant mutants showed inhibited fractalkine-induced cell migration and ICAM-1 expression (Figure 4E-4F). Subsequently, we determined NF-κB promoter activity after fractalkine treatment; MG63 cells were transfected with the κB-luciferase reporter plasmid as a reporter of NF-κB activation. Figure 5A shows that increasing of in κB-luciferase activity after fractalkine treatment. Moreover, pretreatment with the indicated pathway inhibitors (LY294002, wortmannin, Akti, PDTC, and TPCK) or cotransfection with dominant mutants abolished fractalkine-induced NF-κB promoter activity (Figure 5B and 5C). In addition, the PI3K/Akt/NF-κB signal cascade was confirmed by p65 subunit nuclear translocation and phosphorylation. The data revealed that pretreatment with inhibitors of the PI3K/Akt pathway blocked p65 nuclear translocation and phosphorylation (Figure 5D-5E). We examined the binding of NF-κB to its DNA element by using the ChIP assay, which indicated blocking by pathway inhibitors (Figure 5F). According to these results, activation of the NF-κB transcription factor is involved in fractalkine-induced ICAM-1 expression and cell migration. Moreover, the PI3K/Akt/NF-κB signal cascade is responsible for this induction.

### Knockdown of fractalkine reduces metastasis in a mouse model of osteosarcoma

**Figure 6:**
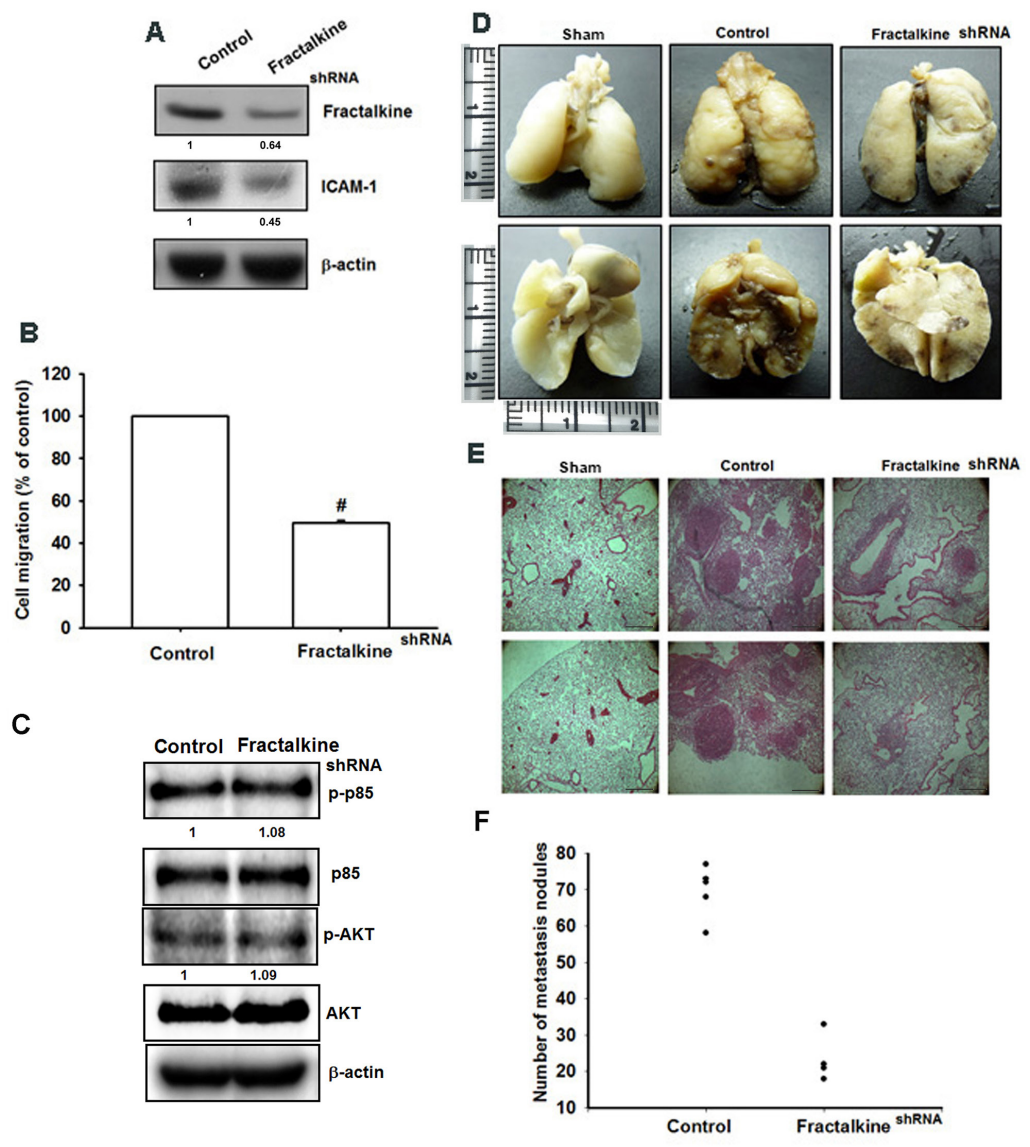
Knockdown of fractalkine inhibits cell migration ability and lung metastasis in osteosarcoma **A**. Total protein was collected from MG63 cells stably expressing fractalkine shRNA. A vector-only control is also shown (control). Western blotting was used to assess the expression of fractalkine and ICAM-1. Actin was used as the loading control. **B**. The cell migration ability of MG63 cells stably expressing a shRNA vector and control vector was measured using the Transwell assay. **C**. The protein levels of p-PI3K, PI3K, p-Akt and Akt in control-shRNA and fractalkine-shRNA cells was examined by western blot. **D**. To induce pulmonary metastases, cells were injected into the mouse tail vein and those mice were sacrificed after 28 days later with developed lung metastatic nodules. Compared to the control mice, there are fewer and smaller tumors which were seen on the lungs of mice injected with osteosarcoma cells transfected with shRNA against fractalkine. **E**. H&E staining of lung metastatic nodules of MG63 injected mice. The scale bars shown on 100× images correspond to 250 μm. **F**. The lungs of MG63 injected mice were removed and inflated with 10 % paraformaldehyde fixative. The number of lung metastatic nodules was counted under a dissecting microscope. Results are shown as mean ± SEM. # represents P < 0.01 fort-test comparisons to cells harboring only the empty vector as control.

The *in vitro* results were confirmed using MG63 cells stably expressing fractalkine shRNA. The expression of fractalkine and ICAM-1 was decreased in clones stably expressing fractalkine shRNA (Figure 6A). As expected, cell migration ability was also decreased in clones stably expressing fractalkine shRNA (Figure 6B). To further confirm that fractalkine mediated ICAM-1-dependent cell migration in human osteosarcoma cells by PI3K-Akt pathway, the expression levels of PI3K and Akt were did not differ between in the sh-fractalkine cells compared with MG63 (Figure 6C). To determine the role of fractalkine in osteosarcoma metastasis *in vivo*, cells were injected into the tail vein, and mice were sacrificed 28 days later. Lung metastasis was identified by counting the number of nodules in tumor-bearing mice, and the data showed that knockdown of fractalkine significantly abolished the mean number of lung metastatic nodules (Figure 6D-6F). Conclusively, knockdown of fractalkine reduced cell migration *in vitro* and lung metastasis *in vivo*.

### Fractalkine expression is associated with ICAM-1 expression in osteosarcoma specimens

**Figure 7:**
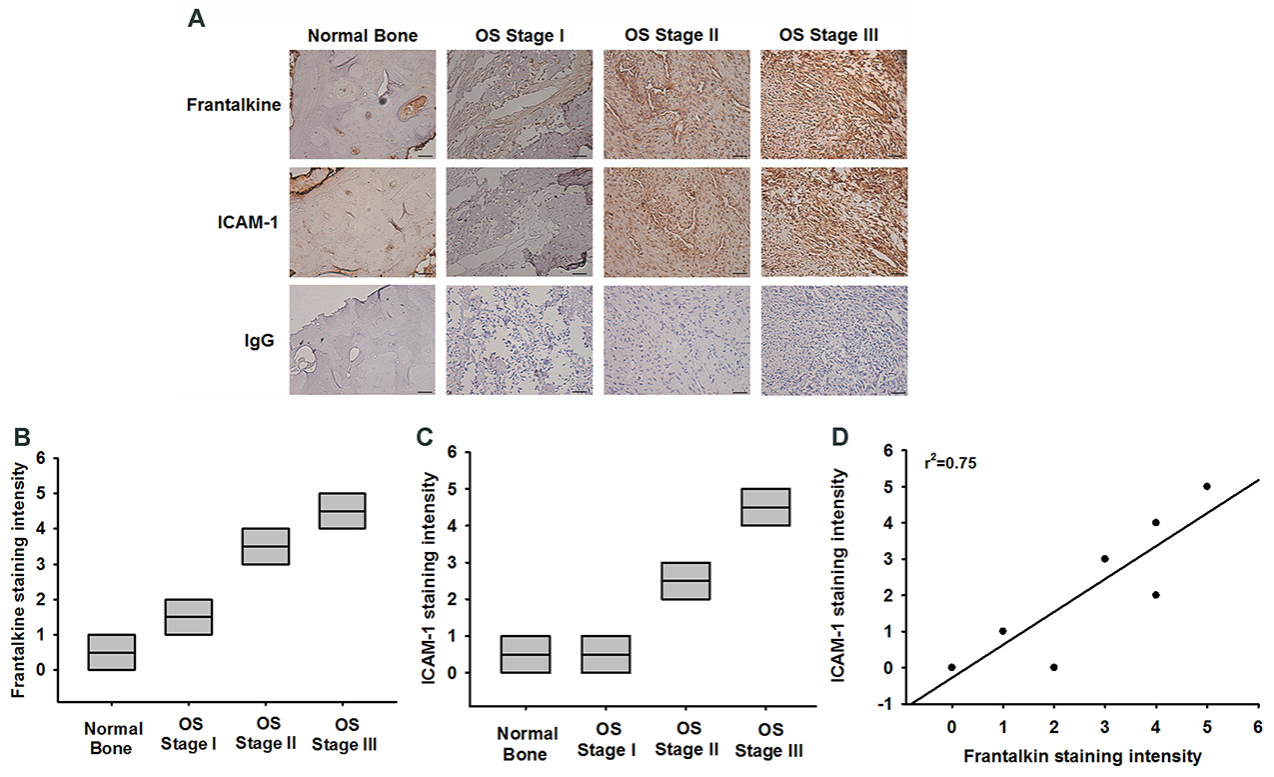
Fractalkine expression is significant correlated with ICAM-1 and tumor stage in osteosarcoma specimens **A**. IHC photographs (Scar bar = 50 μm). The osteosarcoma specimens were stained with fractalkine and ICAM-1 antibodies by IHC staining. **B** and **C**. The staining intensities of fractalkine and ICAM-1 were scored from 1-5 and quantified to show fractalkine and ICAM-1 expression levels in different tumor stages. **D**. Positive correlation between fractalkine and ICAM-1 expression levels in osteosarcoma specimens. Data are expressed as the mean ± SEM.

Finally, it is important to examine the correlation between fractalkine and ICAM-1 in clinical specimens. IHC results showed that fractalkine and ICAM-1 expressions were associated with higher tumor stage (Figure 7A-7C). The staining intensity was evaluated as 0 (negative), 1 (very weak), 2 (weak), 3 (moderate), 4 (strong), and 5 (very strong). Furthermore, fractalkine expression was positively correlated with ICAM-1 expression in osteosarcoma specimens (Figure 7D). These results suggested that fractalkine linked with ICAM-1 and tumor progression in osteosarcoma.

## DISCUSSION

Osteosarcoma is the most common malignant bone tumor in children and young adults. To date, surgery and chemotherapy remain the only treatment strategy, but a mortality rate of 20% due to lung metastasis is still observed. Therefore, novel effective adjuvant therapy for preventing osteosarcoma metastasis is urgently required. This study is the first to provide evidence that fractalkine promotes cell migration and contributes to osteosarcoma metastasis. This effect is mediated through ICAM-1, the expression of which is implicated in the metastasis of many cancers. Our results also demonstrated that CX3CR1/PI3K/Akt/NF-κB mediates ICAM-1 expression, subsequently regulating cell migration and lung metastasis in osteosarcoma. Hence, fractalkine may be a novel cancer therapeutic target.

Factalkine is one of the most expressed chemokines in the brain and has been proposed to have a crucial function in the central nervous system. However, dysregulation of the fractalkine/CX3CR1 axis is correlated with malignancies of the central nervous system, such as gliomas and neuroblastoma [37, 38]. The correlation between the fractalkine/CX3CR1 axis and tumor progression remains controversial. This phenomenon may be caused by the dual function of fractalkine, which functions as a chemoattractant for leukocytes and an adhesion molecule for tumor cells expressing the receptor. For example, high expression of fractalkine is correlated with the number of infiltration lymphocytes and a higher survival rate in patients with colorectal cancer [29]. In neuroblastoma, high expression of fractalkine is also associated with antitumor effects through the regulation of NK cells and lymphocytes [39]. However, fractalkine has protumoral effects for prostate, breast, and pancreatic cancers; high expression of CX3CR1 in these cancers promotes metastasis. This effect may be caused by transmigration of cancer cells through endothelial cells expressing fractalkine [28, 30, 40]. For the first time, our study elucidated the role of the fractalkine/CX3CR1 axis in osteosarcoma, the most common bone tumor in children. Our results indicated that fractalkine expression was positively correlated with tumor progression. High fractalkine expression was responsible for the cell migration and metastasis of osteosarcoma. This study proves the crucial role of fractalkine in bone tumors, consistent with the previous finding that the fractalkine/CX3CR1 axis plays a crucial role in the bone microenvironment of multiple myeloma metastasis [41]. Therefore, the fractalkine/CX3CR1 axis is a potential therapeutic target for treating bone-related tumors.

In this study, the role of ICAM-1 in osteosarcoma metastasis was confirmed. The fractalkine/CX3CR1 axis activated ICAM-1 expression in osteosarcoma and promoted the migration of osteosarcoma cells. ICAM-1 plays a vital role in leukocyte adhesion and cancer cell invasion [42, 43]. Our previous study showed the key role of ICAM-1 in osteosarcoma metastasis [44]. Another study on osteosarcoma revealed that ICAM-1 is upregulated by interleukin-6 and is responsible for the migration of osteosarcoma cells [45]. ICAM-1 also promotes metastasis and is correlated with the progression of other cancers. Knockout of ICAM-1 is associated with decreased spinal metastasis because of a non-organ-specific reduction in metastasis development [46]. In prostate cancer, CCN3 induces ICAM-1 expression and thus promotes bone metastasis [15]. In breast cancer, ICAM-1 expression is positively correlated with tumor progression and poor prognosis [47]. Future studies should investigate the application of the adhesion molecule ICAM-1 to cancer therapy.

PI3K/Akt is the most important signal regulator of survival when cells are exposed to stress [48]. It has been proposed to be a key regulator in tumor initiation and progression, and dysfunction of PI3K/Akt has been observed in several tumors [49]. Therefore, the PI3K/Akt pathway has implications in cancer drug discovery and development [50]. Many studies have demonstrated PI3K/Akt activation by the fractalkine/CX3CR1 axis. In multiple myeloma, fractalkine induces Akt activation and cell adhesion through CX3CR1 [41]. In chronic lymphocytic leukemia, the fractalkine/CX3CR1 axis activates Akt and is involved in the interaction of chronic lymphocytic leukemia with the microenvironment [51]. Moreover, in neuroblastoma, the fractalkine/CX3CR1 axis promotes transendothelial migration and contributes to bone marrow metastasis through Akt activation [38]. In our present study, we confirmed that the PI3K/Akt signal cascade is involved in fractalkine/CX3CR1-induced ICAM-1 expression and lung metastasis in osteosarcoma. Our results revealed that PI3K/Akt may be a crucial signal regulator mediating fractalkine/CX3CR1 signal transduction in various cancers.

For the first time, the role of the fractalkine/CX3CR1 axis was elucidated in this study. Our findings indicate that the fractalkine/CX3CR1 axis is correlated with tumor progression and promotes cell migration in osteosarcoma. fractalkine/CX3CR1-induced ICAM-1 expression and cell migration are mediated by the PI3K/Akt/NF-κB signaling pathway. In conclusion, we provide evidence that fractalkine expression is associated with osteosarcoma metastasis, indicating that fractalkine is a novel therapeutic target for preventing osteosarcoma metastasis.

## MATERIALS AND METHODS

### Materials

Anti-mouse and anti-rabbit IgG-conjugated horseradish peroxidase and rabbit polyclonal antibodies specific for fractalkine/CX3CL1, CX3CR1, ICAM-1, VCAM-1, p-PI3K p85α, PI3K p85α, p-Akt, Akt, p-IKKα/β, IKKα/β, p-IκBα, IκBα, p-p65, p65, and β-actin were purchased from Santa Cruz Biotechnology (Santa Cruz, CA, USA). Recombinant human fractalkine/CX3CL1 was purchased from PeproTech (Rocky Hill, NJ, USA). The short hairpin RNA (shRNA) plasmid used for gene knockdown was purchased from the National RNAi Core Facility Platform (Taipei, Taiwan). All siRNAs used were ON-TARGETplus siRNAs and purchased from Dharmacon Research (Lafayette, CO, USA). All other chemicals were obtained from Sigma–Aldrich (St. Louis, MO, USA).

### Cell culture

The human osteosarcoma cell lines MG63, U2OS and the human osteoblast cell line hFOB 1.19 were purchased from the American Type Cell Culture Collection (Manassas, VA, USA). All cell lines were maintained in DMEM supplemented with 20 mM HEPES, 10% heat-inactivated fetal bovine serum, 2 mM glutamine, 100 U/mL penicillin, and 100 μg/mL streptomycin at 37°C with 5% CO_2_. hFOB 1.19 cells were maintained under the same condition but at 33°C.

To generate fractalkine stable knockdown cell line of MG63 cells, the cells were transfected with fractalkine shRNA by using Lipofectamine 2000 (Invitrogen, Carlsbad, CA, USA), and fractalkine shRNA-expressing MG63 cells were selected using puromycin (1 μg/mL). The selected cells were expanded to generate clonal cell populations.

### Western blotting

The cells were lysed in a RIPA lysis buffer, and total cell lysates were collected. Proteins were resolved through SDS-PAGE and transferred to an Immobilon polyvinyldifluoride membrane. Blots were blocked with 5% nonfat milk for 1 h at room temperature. The blots were incubated with antibodies (1:1000) for 1 h at room temperature. After 3 washes, the blots were incubated with a peroxidase-conjugated secondary antibody (1:1000) for 1 h at room temperature and visualized using enhanced chemiluminescence with ImageQuant™ LAS 4000 (GE Healthcare Life Sciences, Little Chalfont, UK). The developed bands were quantified by using a computing densitometer and ImageQuant software.

### Quantitative real-time PCR

qPCR was performed using the Taqman® one-step PCR Master Mix (Applied Biosystems, Foster City, CA, USA). Total cDNA (100 ng) was added per 25-μL reaction with sequence-specific primers and Taqman® probes. All target gene primers and probes were purchased from Applied Biosystems (β-actin was the internal control); the qPCR assay was triplicated using the StepOnePlus sequence detection system. Cycling conditions were as follows: polymerase activation at 95°C for 10 min, followed by 40 cycles at 95°C for 15 s and 60°C for 60 s; the threshold was set above a nontemplate control background and within the linear phase of target gene amplification to calculate the cycle number at which the transcript was detected (denoted as CT).

### Transwell cell migration assay

A cell migration assay was performed using Transwell inserts (8-μm pore size; Costar, NY, USA) in 24-well dishes. The cells were pretreated for 30 min with the designated inhibitor and then incubated with fractalkine for 24 h. The cells were seeded in the upper Transwell chamber, and 300 μL of a medium was added to the lower chamber. After 24 h, the cells were fixed in 3.7% formaldehyde for 15 min and stained with 0.05% crystal violet for 15 min. Cells on the upper side of the chamber were removed using cotton-tipped swabs, and the chamber was washed with phosphate-buffered saline (PBS). The cells on the underside of the filters were examined and counted under a microscope. Each experiment was repeated at least 3 times.

### Measurement of soluble levels of fractalkine

Fractalkine concentration was measured in culture supernatants with Human Fractalkine DuoSet ELISA (R&D Systems in accordance with the manufacturer's protocol. All samples were stored at −80°C before use. The samples were assayed in duplicate.

### Flow cytometric analysis

MG63 osteosarcoma cells were prepared under the indicated condition in 6-well plates and then washed with PBS and detached using trypsin (Gibco, CA, USA) at 37°C. The cells were fixed for 10 min in 3.7% paraformaldehyde, rinsed with PBS, and incubated with mouse anti-human-ICAM-1 (1:100) (BD Biosciences, CA, USA) for 1 h at room temperature. After incubation, the cells were washed 3 times with PBS and then incubated with FITC-conjugated goat antimouse secondary IgG (1:100; Leinco Technologies, St. Louis, MO, USA) for 45 min at room temperature. After a final rinse, the cells were analyzed using an FACSCalibur flow cytometer and CellQuest software (BD Biosciences).

### Immunofluorescence microscopy

MG63 cells were seeded on glass coverslips and treated under the indicated conditions; the cells were rinsed once with PBS and fixed in 3.7% paraformaldehyde for 10 min at room temperature. The cells were washed 3 times with PBS and blocked with 4% BSA for 15 min. Subsequently, the cells were incubated with antihuman p65 (1:100) for 1 h at room temperature. After being washed 3 times, the cells were incubated with FITC-conjugated goat anti-rabbit IgG for 1 h. Finally, the cells were washed, mounted, and photographed using the Leica TCS SP2 Spectral Confocal System.

### Chromatin immunoprecipitation assay

A chromatin immunoprecipitation (ChIP) assay was performed as described previously [52]. DNA immunoprecipitated by the anti-p65 antibody was purified. DNA was then extracted using phenol–chloroform. The purified DNA pellet was subjected to PCR. PCR products were then resolved through 1.5% agarose gel electrophoresis and visualized using ultraviolet irradiation. The primers 5’-AGACCTTAGCG CGGTGTAGA-3’ and 5’-AGTAGCAGAGGAGCTCAGCG-3’ were used to amplify DNA segments across the ICAM-1 promoter region (−346 to −24).

### Reporter assay

A NF-κB report plasmid, pSV-β-galactosidase vector, and luciferase assay kit were purchased from Promega (Madison, WI, USA). The cells were cotransfected with the NF-κB report plasmid (0.7 μg) and the pSV-β-galactosidase vector (0.3 μg) for 24 h by using Lipofectamine 3000 (Invitrogen). According to the manufacturer's recommendations, cell extracts were prepared, and luciferase and β-galactosidase activities were then measured.

### In vivo tumor xenograft study

Four-week-old male SCID mice were purchased from Lasco (Taipei, Taiwan) and were maintained in pathogen-free conditions. All animal experiments were performed in accordance with the protocol approved by the Institutional Animal Care and Use Committees of Shin Kong Wu Ho-Su Memorial Hospital (Taipei, Taiwan). Seven animals per group, were used, and the experiment was repeated twice. For assessing lung metastasis in osteosarcoma cells in the *in vivo* xenograft model, 1 × 10^6^ cells were resuspended in 100 μL of PBS and injected into the lateral tail vein. After 4 weeks, the mice were sacrificed, and the lungs were removed and fixed in 10% formaldehyde. The number of metastatic nodules in the lungs was counted under a dissecting microscope.

### Immunohistochemistry (IHC)

Human normal bone and osteosarcoma tissue microarray (BO244, T261, T262, T262A, T263 and OS804b), containing 74 cases of 11 case of normal bone, 7 case of Stage I osteosarcoma, 49 case of Stage II osteosarcoma and 7 case of Stage III osteosarcoma, were purchased from Biomax (Rockville, MD). Sections (5-μm thick) of paraffin-embedded tissue were placed on glass slides, rehydrated, incubated with 3% hydrogen peroxide to quench endogenous peroxidase activity, then blocked by 3% BSA incubation in PBS. Sections were incubated with the primary mouse polyclonal anti-human fractalkine and ICAM-1 antibody at 1:100 dilutions and incubated at 4°C overnight. After three PBS washes, samples were incubated with a 1:100 dilution of biotin-labeled goat anti-mouse IgG secondary antibody, bound antibodies detected by ABC Kit (Vector Laboratories, Burlingame, CA). Slides were stained with chromogen diaminobenzidine, washed, counterstained with Delafield's hematoxylin, dehydrated, treated with xylene, then mounted. The staining intensity was evaluated as 0 (negative ), 1 (very weak), 2 (weak), 3 (moderate), 4 (strong), and 5 (very strong), respectively, by five independent and blinded observers. IHC score was determined as the sum of the intensity score.

### Statistical analysis

Data are presented as the mean ± standard error of the mean (SEM). Statistical comparisons between 2 groups were performed using the Student's *t* test. Statistical comparisons of more than 2 groups were performed using one-way analysis of variance with Bonferroni's post hoc test. A P value of less than 0.05 was considered statistically significant.

## SUPPLEMENTARY MATERIALS FIGURES



## References

[1] Hansen MF, Seton M, Merchant A (2006). Osteosarcoma in Paget's disease of bone. J Bone Miner Res.

[2] Ottaviani G, Jaffe N (2009). The etiology of osteosarcoma. Cancer Treat Res.

[3] Kempf-Bielack B, Bielack SS, Jurgens H, Branscheid D, Berdel WE, Exner GU, Gobel U, Helmke K, Jundt G, Kabisch H, Kevric M, Klingebiel T, Kotz R (2005). Osteosarcoma relapse after combined modality therapy: an analysis of unselected patients in the Cooperative Osteosarcoma Study Group (COSS). Journal of clinical oncology.

[4] Arndt CA, Crist WM (1999). Common musculoskeletal tumors of childhood and adolescence. The New England journal of medicine.

[5] Bacci G, Ferrari S, Longhi A, Perin S, Forni C, Fabbri N, Salduca N, Versari M, Smith KV (2001). Pattern of relapse in patients with osteosarcoma of the extremities treated with neoadjuvant chemotherapy. Eur J Cancer.

[6] Juliano R (1994). Signal transduction by integrins and its role in the regulation of tumor growth. Cancer metastasis reviews.

[7] Yamamoto H, Irie A, Fukushima Y, Ohnishi T, Arita N, Hayakawa T, Sekiguchi K (1996). Abrogation of lung metastasis of human fibrosarcoma cells by ribozyme-mediated suppression of integrin alpha6 subunit expression. International journal of cancer.

[8] Aoudjit F, Potworowski EF, Springer TA, St-Pierre Y (1998). Protection from lymphoma cell metastasis in ICAM-1 mutant mice: a posthoming event. J Immunol.

[9] Zimmerman T, Blanco FJ (2008). Inhibitors targeting the LFA-1/ICAM-1 cell-adhesion interaction: design and mechanism of action. Current pharmaceutical design.

[10] Lawson C, Wolf S (2009). ICAM-1 signaling in endothelial cells. PR.

[11] van de Stolpe A, van der Saag PT (1996). Intercellular adhesion molecule-1. J Mol Med (Berl).

[12] Yang SF, Chen MK, Hsieh YS, Chung TT, Hsieh YH, Lin CW, Su JL, Tsai MH, Tang CH (2010). Prostaglandin E2/EP1 signaling pathway enhances intercellular adhesion molecule 1 (ICAM-1) expression and cell motility in oral cancer cells. J Biol Chem.

[13] Rauch BH, Muschenborn B, Braun M, Weber AA, Schror K (2007). ICAM-1 and p38 MAPK mediate fibrinogen-induced migration of human vascular smooth muscle cells. European journal of pharmacology.

[14] Roche Y, Pasquier D, Rambeaud JJ, Seigneurin D, Duperray A (2003). Fibrinogen mediates bladder cancer cell migration in an ICAM-1-dependent pathway. Thrombosis and haemostasis.

[15] Chen PC, Lin TH, Cheng HC, Tang CH (2012). CCN3 increases cell motility and ICAM-1 expression in prostate cancer cells. Carcinogenesis.

[16] Kuai WX, Wang Q, Yang XZ, Zhao Y, Yu R, Tang XJ (2012). Interleukin-8 associates with adhesion, migration, invasion and chemosensitivity of human gastric cancer cells. WJG.

[17] Chen LM, Kuo CH, Lai TY, Lin YM, Su CC, Hsu HH, Tsai FJ, Tsai CH, Huang CY, Tang CH (2011). RANKL increases migration of human lung cancer cells through intercellular adhesion molecule-1 up-regulation. J Cell Biochem.

[18] Fong YC, Lin CY, Su YC, Chen WC, Tsai FJ, Tsai CH, Huang CY, Tang CH (2012). CCN6 enhances ICAM-1 expression and cell motility in human chondrosarcoma cells. Journal of cellular physiology.

[19] Miele ME, Bennett CF, Miller BE, Welch DR (1994). Enhanced metastatic ability of TNF-alpha-treated malignant melanoma cells is reduced by intercellular adhesion molecule-1 (ICAM-1, CD54) antisense oligonucleotides. Experimental cell research.

[20] Zhu XW, Gong JP (2013). Expression and role of icam-1 in the occurrence and development of hepatocellular carcinoma. APJCP.

[21] Bazan JF, Bacon KB, Hardiman G, Wang W, Soo K, Rossi D, Greaves DR, Zlotnik A, Schall TJ (1997). A new class of membrane-bound chemokine with a CX3C motif. Nature.

[22] Muehlhoefer A, Saubermann LJ, Gu X, Luedtke-Heckenkamp K, Xavier R, Blumberg RS, Podolsky DK, MacDermott RP, Reinecker HC (2000). Fractalkine is an epithelial and endothelial cell-derived chemoattractant for intraepithelial lymphocytes in the small intestinal mucosa. J Immunol.

[23] Fong AM, Robinson LA, Steeber DA, Tedder TF, Yoshie O, Imai T, Patel DD (1998). Fractalkine and CX3CR1 mediate a novel mechanism of leukocyte capture, firm adhesion, and activation under physiologic flow. J Exp Med.

[24] Ludwig A, Berkhout T, Moores K, Groot P, Chapman G (2002). Fractalkine is expressed by smooth muscle cells in response to IFN-gamma and TNF-alpha and is modulated by metalloproteinase activity. J Immunol.

[25] Papadopoulos EJ, Sassetti C, Saeki H, Yamada N, Kawamura T, Fitzhugh DJ, Saraf MA, Schall T, Blauvelt A, Rosen SD, Hwang ST (1999). Fractalkine, a CX3C chemokine, is expressed by dendritic cells and is up-regulated upon dendritic cell maturation. Eur J Immunol.

[26] Yoshikawa M, Nakajima T, Matsumoto K, Okada N, Tsukidate T, Iida M, Otori N, Haruna S, Moriyama H, Imai T, Saito H (2004). TNF-alpha and IL-4 regulate expression of fractalkine (CX3CL1) as a membrane-anchored proadhesive protein and soluble chemotactic peptide on human fibroblasts. FEBS Lett.

[27] Imai T, Hieshima K, Haskell C, Baba M, Nagira M, Nishimura M, Kakizaki M, Takagi S, Nomiyama H, Schall TJ, Yoshie O (1997). Identification and molecular characterization of fractalkine receptor CX3CR1, which mediates both leukocyte migration and adhesion. Cell.

[28] Andre F, Cabioglu N, Assi H, Sabourin JC, Delaloge S, Sahin A, Broglio K, Spano JP, Combadiere C, Bucana C, Soria JC, Cristofanilli M (2006). Expression of chemokine receptors predicts the site of metastatic relapse in patients with axillary node positive primary breast cancer. Ann Oncol.

[29] Ohta M, Tanaka F, Yamaguchi H, Sadanaga N, Inoue H, Mori M (2005). The high expression of Fractalkine results in a better prognosis for colorectal cancer patients. Int J Oncol.

[30] Shulby SA, Dolloff NG, Stearns ME, Meucci O, Fatatis A (2004). CX3CR1-fractalkine expression regulates cellular mechanisms involved in adhesion, migration, and survival of human prostate cancer cells. Cancer Res.

[31] Hyakudomi M, Matsubara T, Hyakudomi R, Yamamoto T, Kinugasa S, Yamanoi A, Maruyama R, Tanaka T (2008). Increased expression of fractalkine is correlated with a better prognosis and an increased number of both CD8+ T cells and natural killer cells in gastric adenocarcinoma. Annals of surgical oncology.

[32] Li F, Wang Z, Liu Y, Li J (2010). Down-regulation of fractalkine inhibits the in vitro and in vivo angiogenesis of the hepatocellular carcinoma HepG2 cells. Oncol Rep.

[33] Erreni M, Solinas G, Brescia P, Osti D, Zunino F, Colombo P, Destro A, Roncalli M, Mantovani A, Draghi R, Levi D, Rodriguez YBR, Gaetani P (2010). Human glioblastoma tumours and neural cancer stem cells express the chemokine CX3CL1 and its receptor CX3CR1. Eur J Cancer.

[34] Gaudin F, Nasreddine S, Donnadieu AC, Emilie D, Combadiere C, Prevot S, Machelon V, Balabanian K (2011). Identification of the chemokine CX3CL1 as a new regulator of malignant cell proliferation in epithelial ovarian cancer. PloS one.

[35] Schlesinger M, Bendas G (2015). Vascular cell adhesion molecule-1 (VCAM-1)--an increasing insight into its role in tumorigenicity and metastasis. Int J Cancer.

[36] Tang CH, Tan TW, Fu WM, Yang RS (2008). Involvement of matrix metalloproteinase-9 in stromal cell-derived factor-1/CXCR4 pathway of lung cancer metastasis. Carcinogenesis.

[37] Sciume G, Soriani A, Piccoli M, Frati L, Santoni A, Bernardini G (2010). CX3CR1/CX3CL1 axis negatively controls glioma cell invasion and is modulated by transforming growth factor-beta1. Neuro-oncology.

[38] Nevo I, Sagi-Assif O, Meshel T, Ben-Baruch A, Johrer K, Greil R, Trejo LE, Kharenko O, Feinmesser M, Yron I, Witz IP (2009). The involvement of the fractalkine receptor in the transmigration of neuroblastoma cells through bone-marrow endothelial cells. Cancer Lett.

[39] Zeng Y, Jiang J, Huebener N, Wenkel J, Gaedicke G, Xiang R, Lode HN (2005). Fractalkine gene therapy for neuroblastoma is more effective in combination with targeted IL-2. Cancer Lett.

[40] Marchesi F, Piemonti L, Fedele G, Destro A, Roncalli M, Albarello L, Doglioni C, Anselmo A, Doni A, Bianchi P, Laghi L, Malesci A, Cervo L (2008). The chemokine receptor CX3CR1 is involved in the neural tropism and malignant behavior of pancreatic ductal adenocarcinoma. Cancer Res.

[41] Wada A, Ito A, Iitsuka H, Tsuneyama K, Miyazono T, Murakami J, Shibahara N, Sakurai H, Saiki I, Nakayama T, Yoshie O, Koizumi K, Sugiyama T (2015). Role of chemokine CX3CL1 in progression of multiple myeloma via CX3CR1 in bone microenvironments. Oncology reports.

[42] Maruo Y, Gochi A, Kaihara A, Shimamura H, Yamada T, Tanaka N, Orita K (2002). ICAM-1 expression and the soluble ICAM-1 level for evaluating the metastatic potential of gastric cancer. International journal of cancer.

[43] Liu G, Place AT, Chen Z, Brovkovych VM, Vogel SM, Muller WA, Skidgel RA, Malik AB, Minshall RD (2012). ICAM-1-activated Src and eNOS signaling increase endothelial cell surface PECAM-1 adhesivity and neutrophil transmigration. Blood.

[44] Hou CH, Lin FL, Tong KB, Hou SM, Liu JF (2014). Transforming growth factor alpha promotes osteosarcoma metastasis by ICAM-1 and PI3K/Akt signaling pathway. Biochem Pharmacol.

[45] Lin YM, Chang ZL, Liao YY, Chou MC, Tang CH (2013). IL-6 promotes ICAM-1 expression and cell motility in human osteosarcoma. Cancer Lett.

[46] Broggini T, Czabanka M, Piffko A, Harms C, Hoffmann C, Mrowka R, Wenke F, Deutsch U, Grotzinger C, Vajkoczy P (2015). ICAM1 depletion reduces spinal metastasis formation in vivo and improves neurological outcome. European spine journal.

[47] Schroder C, Witzel I, Muller V, Krenkel S, Wirtz RM, Janicke F, Schumacher U, Milde-Langosch K (2011). Prognostic value of intercellular adhesion molecule (ICAM)-1 expression in breast cancer. J Cancer Res Clin Oncol.

[48] Datta SR, Brunet A, Greenberg ME (1999). Cellular survival: a play in three Akts. Genes Dev.

[49] Lim HJ, Crowe P, Yang JL (2015). Current clinical regulation of PI3K/PTEN/Akt/mTOR signalling in treatment of human cancer. J Cancer Res Clin Oncol.

[50] Garcia-Echeverria C, Sellers WR (2008). Drug discovery approaches targeting the PI3K/Akt pathway in cancer. Oncogene.

[51] Ferretti E, Bertolotto M, Deaglio S, Tripodo C, Ribatti D, Audrito V, Blengio F, Matis S, Zupo S, Rossi D, Ottonello L, Gaidano G, Malavasi F (2011). A novel role of the CX3CR1/CX3CL1 system in the cross-talk between chronic lymphocytic leukemia cells and tumor microenvironment. Leukemia.

[52] Chiu YC, Yang RS, Hsieh KH, Fong YC, Way TD, Lee TS, Wu HC, Fu WM, Tang CH (2007). Stromal cell-derived factor-1 induces matrix metalloprotease-13 expression in human chondrocytes. Mol Pharmacol.

