# Supplementary material for: Fractalkine/CX3CL1 induced intercellular adhesion molecule-1-dependent tumor metastasis through the CX3CR1/PI3K/Akt/NF-κB pathway in human osteosarcoma
Source: Oncotarget. 2016 Aug 12;8(33):54136–48. doi: 10.18632/oncotarget.11250 (PMC5589568; doi:10.18632/oncotarget.11250)
Supplement: Supplementary file 1 [file oncotarget-08-54136-s001.pdf]

Fractalkine/CX3CL1 induced intercellular adhesion molecule-1-dependent tumor metastasis through the CX3CR1/PI3K/Akt/NF-κB pathway in human osteosarcoma

SUPPLEMENTARY FIGURE

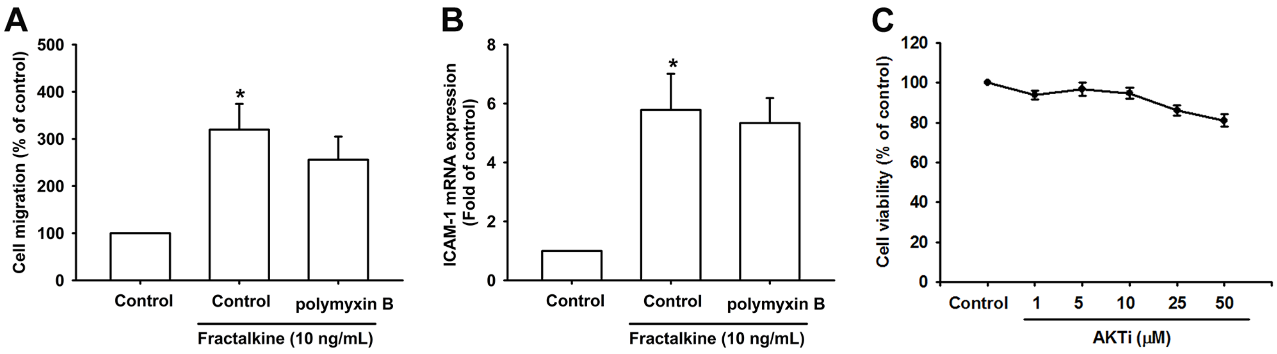

Supplementary Figure S1: A-B. MG63 cells were pretreated with polymyxin B (poly B, 1 μM) for 30 min followed by stimulation with fractalkine (10 ng/ml), the cell migration and ICAM-1 expression were examined by the Transwell migration assay and qPCR. **C.** MG63 cells were incubated with serial concentrations of AKTi (0-50 μM) for 48h, and cell viability was examined using an MTT assay. Results are expressed as the mean ± SEM of triplicate samples. *P < 0.05 compared with the control group.
